# Quantitative intravital imaging for real-time monitoring of pancreatic tumor cell hypoxia and stroma in an orthotopic mouse model

**DOI:** 10.1126/sciadv.ade8672

**Published:** 2023-06-07

**Authors:** Timothy Samuel, Sara Rapic, Cristiana O’Brien, Michael Edson, Yuan Zhong, Ralph S. DaCosta

**Affiliations:** ^1^ Princess Margaret Cancer Centre, University Health Network, Toronto, Canada.; ^2^ Department of Medical Biophysics, University of Toronto, Toronto, Canada.

## Abstract

Pancreatic cancer is a lethal disease with few successful treatment options. Recent evidence demonstrates that tumor hypoxia promotes pancreatic tumor invasion, metastasis, and therapy resistance. However, little is known about the complex relationship between hypoxia and the pancreatic tumor microenvironment (TME). In this study, we developed a novel intravital fluorescence microscopy platform with an orthotopic mouse model of pancreatic cancer to study tumor cell hypoxia within the TME in vivo, at cellular resolution, over time. Using a fluorescent BxPC3-DsRed tumor cell line with a hypoxia-response element (HRE)/green fluorescent protein (GFP) reporter, we showed that HRE/GFP is a reliable biomarker of pancreatic tumor hypoxia, responding dynamically and reversibly to changing oxygen concentrations within the TME. We also characterized the spatial relationships between tumor hypoxia, microvasculature, and tumor-associated collagen structures using in vivo second harmonic generation microscopy. This quantitative multimodal imaging platform enables the unprecedented study of hypoxia within the pancreatic TME in vivo.

## Teaser

Live-animal fluorescence microscopy was used to study hypoxia within the pancreatic tumor microenvironment.

## INTRODUCTION

Despite decades of research, the current standard of care for pancreatic cancer, on average, provides only a few months of survival benefit (*1*, *2*). The poor outcomes associated with this disease are due in combination to its late diagnosis (*3*) as well as both inherent and acquired treatment resistance (*4*). Pancreatic tumors tend to be resistant to both chemo- and radiotherapy (*5*–*7*), as well as more contemporary immunotherapies (*8*).

The complex pancreatic tumor microenvironment (TME), characterized by hypoxia and desmoplasia, plays a crucial role in treatment response (*9*–*13*). As cancer cells proliferate and invade the surrounding tissues, they develop a pronounced stromal compartment, made up of dense fibrotic tissue (*14*). Proliferating fibroblasts secrete extracellular matrix (ECM) proteins [primarily type I collagen; (*15*)], which can act as a physical barrier to treatments (*16*) and cause disruption of vascular networks, leading to impaired blood perfusion and the development and exacerbation of tumor hypoxia (*16*–*20*). Hypoxia up-regulates hypoxia-inducible factors (HIFs) in tumor cells that, besides promoting cancer progression, invasion, and metastasis (*21*), can further stimulate collagen deposition (*22*, *23*), creating a positive feedback loop and the emergence of a more invasive phenotype (*24*).

These characteristics not only make pancreatic cancer difficult to treat but also pose several challenges in studying the development, progression, and treatment of pancreatic tumors in vivo (*25*–*27*). While major advancements in genetically engineered mouse models (GEMMs) (*28*, *29*) and genomics (*30*) have been used to improve our knowledge of pancreatic tumor biology, current preclinical research methodologies are still limited in their ability to study the dynamic changes of the pancreatic TME in the in vivo, orthotopic setting. Conventional murine–based studies of pancreatic cancer largely rely on resected tumor tissue specimens collected at fixed time points from euthanized mice. While such an approach enables detailed examination of histological and immunohistological tumor features and their spatial variation under various experimental conditions, it lacks temporal information within the same mouse. This experimental limitation inhibits our understanding of the biological and treatment-induced spatiotemporal changes affecting pancreatic tumors, as well as their supporting vasculature and TME. Previously, some approaches have used ultrasound and magnetic resonance imaging to study changes in bulk properties, e.g., tumor volume and perfusion, of pancreatic tumors in the same animal serially over time (*31*). However, these methods lack the spatial resolution required to visualize the distinct heterogeneity of tumor cells, stroma, microvasculature, and hypoxia in pancreatic tumors at a cellular level in vivo.

To overcome these challenges, we designed and surgically implanted a pancreatic imaging window (PIW) to perform intravital fluorescence microscopy (IVFM) in an orthotopic mouse model of pancreatic cancer. Using this technique, we were able to directly image pancreatic cancer cells and their TME at cellular resolution, within the same animal, for up to 4 weeks. Using a dually fluorescent human pancreatic cancer cell line, we demonstrate that it is possible to visualize DsRed fluorescent pancreatic tumor cells together with a HIF-driven green fluorescent protein (GFP) reporter in vivo and quantitatively validate this GFP fluorescence as an in vivo biomarker of pancreatic tumor cell hypoxia. We also use allophycocyanin (APC)–conjugated anti-CD31 fluorescent dye and second harmonic generation (SHG) microscopy to simultaneously image tumor microvasculature and collagen structures in the stromal compartment and quantitatively characterize their spatial relationship with tumor cell HIF activity in vivo. Overall, our findings elucidate an intricate relationship between tumor hypoxia, microvasculature, and collagen structures within the pancreatic TME. Future studies with our in vivo IVFM model can be designed to further explore these complex relationships sustaining pancreatic tumor development and explore mechanisms of treatment resistance to help develop more effective treatment strategies and improve outcomes of pancreatic cancer.

## RESULTS

### BxPC3 5xHRE/GFP expression is driven by hypoxia in vitro

**Fig. 1.**
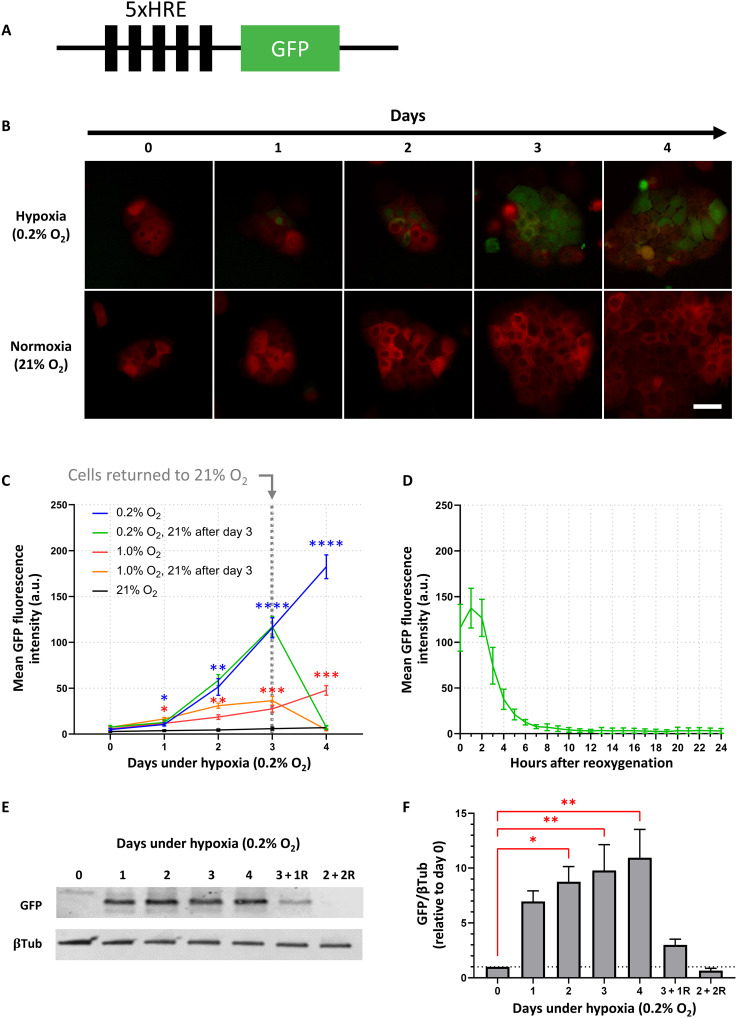
Hypoxia drives 5xHRE-induced GFP expression in BxPC3-DsRed-5xHRE/GFP cells. (**A**) Schematic of 5xHRE/GFP construct. (**B**) Representative images of BxPC3-DsRed-5xHRE/GFP cells growing under hypoxia (0.2% O_2_) and normoxia (21% O_2_) using in vitro live-cell fluorescence microscopy. Scale bar, 50 μm. (**C**) Quantification of GFP fluorescence intensity at 0.2, 1, and 21% O_2_. The green and orange lines indicate cells returned to 21% O_2_ between days 3 and 4. *n* = 9 replicates per group. (**D**) Hourly time lapse of the mean GFP fluorescence intensity after BxPC3-DsRed-5xHRE/GFP cells (previously incubated under 0.2% O_2_ for 3 days) were transferred to 21% O_2_ confirms the oxygen dependence of 5xHRE/GFP construct. *n* = 9 replicates per group. a.u., arbitrary units. (**E**) Western blot and (**F**) densitometric analysis of GFP expression [relative to β-tubulin (βTub)] after various time durations under 0.2% O_2_. Columns labeled with letter “R” indicate the number of days cells were reoxygenated at 21% O_2_ before protein collection. Data were obtained from three independent experiments. *P < 0.05, **P < 0.01, ***P < 0.001, and ****P < 0.0001.

In vitro live-cell fluorescence microscopy was performed daily (for up to 4 days) to investigate the time course of BxPC3 tumor cell 5xHRE (5x hypoxia-response element)/GFP expression under 0.2, 1, and 21% O_2_. We found that, for both 0.2 and 1% O_2_, tumor cell GFP expression rose gradually over the course of 4 days, with the fastest rise occurring under 0.2% O_2_ (Fig. 1C). Cells that were returned to normoxia (21% O_2_) after 3 days in 0.2% O_2_ returned to baseline GFP fluorescence within 1 day. By performing hourly time-lapse imaging on cells that were kept under 0.2% O_2_ for 3 days and subsequently returned to 21% O_2_, we found that tumor cell GFP fluorescence intensity had a half-life of approximately ~2 hours, returning to baseline values within 8 to 12 hours (Fig. 1D). Because cellular GFP synthesis is known to be oxygen dependent (*32*), Western blots were also performed to confirm GFP expression (Fig. 1E). Quantitative densitometric analysis of BxPC3-DsRed-5xHRE/GFP cell lysates demonstrated a similar trend, with GFP expression rising gradually over the course of 4 days under 0.2% O_2_ (Fig. 1F). However, for cells that were kept under 0.2% O_2_ for 3 days and subsequently returned to 21% O_2_ for 1 day (“3 + 1R”), GFP was still detected (although not statistically significant, *P* = 0.84). Only after exposing cells to 2 days under 0.2% O_2_ followed by 2 days under 21% O_2_ (“2 + 2R”) did GFP expression return to baseline levels (*P* > 0.99).

### In vivo BxPC3 5xHRE/GFP fluorescence increases with distance from tumor blood vessels

**Fig. 2.**
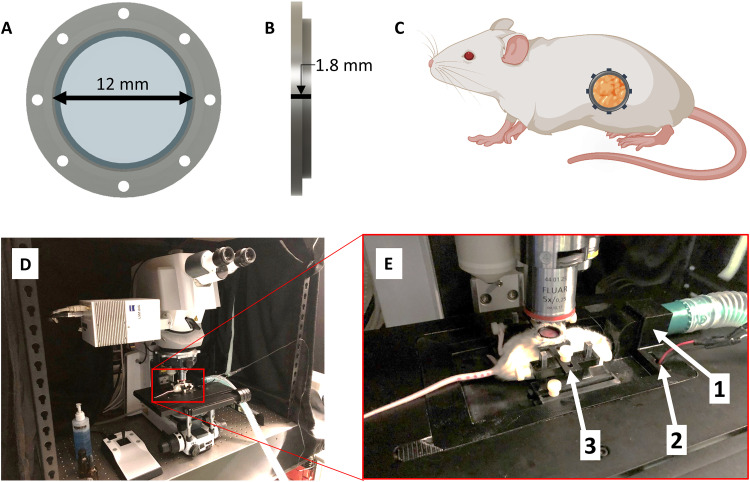
Surgically implanted abdominal imaging window allows longitudinal intravital imaging of pancreatic tumors in vivo. (**A** and **B**) Computer-aided design of an abdominal PIW frame holding a circular glass coverslip (12 mm diameter). (**C**) Diagram of a mouse (using BioRender.com) indicating the anatomical location of the surgically implanted PIW, providing longitudinal imaging access to the pancreas in vivo. (**D**) Photo of the laser scanning confocal microscope with (**E**) a custom-designed, three-dimensionally (3D) printed stage insert to stabilize the animal for anesthesia and imaging. The stage is equipped with (1) a gas anesthesia port, (2) an electrical heating element, and (3) a holder to minimize motion artifacts during imaging and produce consistent images.

**Fig. 3.**
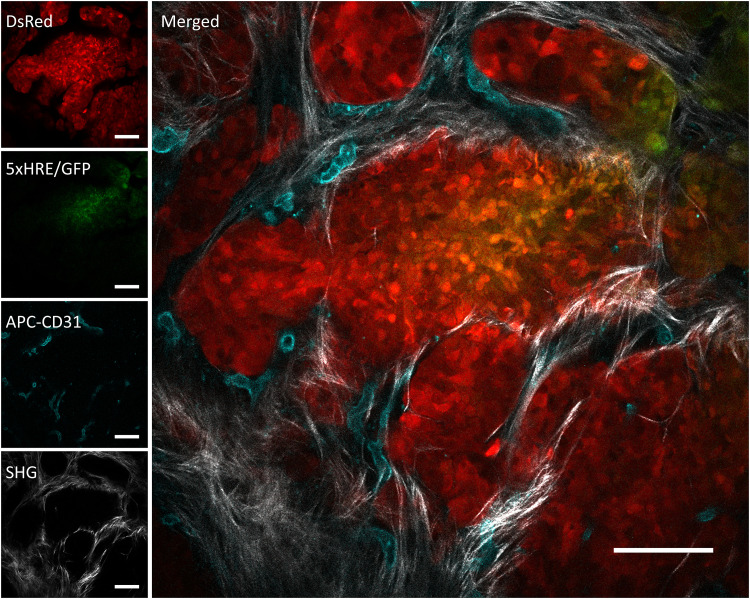
A representative image of an in vivo orthotopic BxPC3-DsRed-5xHRE/GFP tumor foci, taken using intravital fluorescence and SHG microscopy. BxPC3 cells (DsRed) are shown in red, HIF activity (5xHRE/GFP) in green, blood vessels (APC-CD31) in cyan, and fibrillar collagen (SHG) in white. Scale bars, 100 μm.

**Fig. 4.**
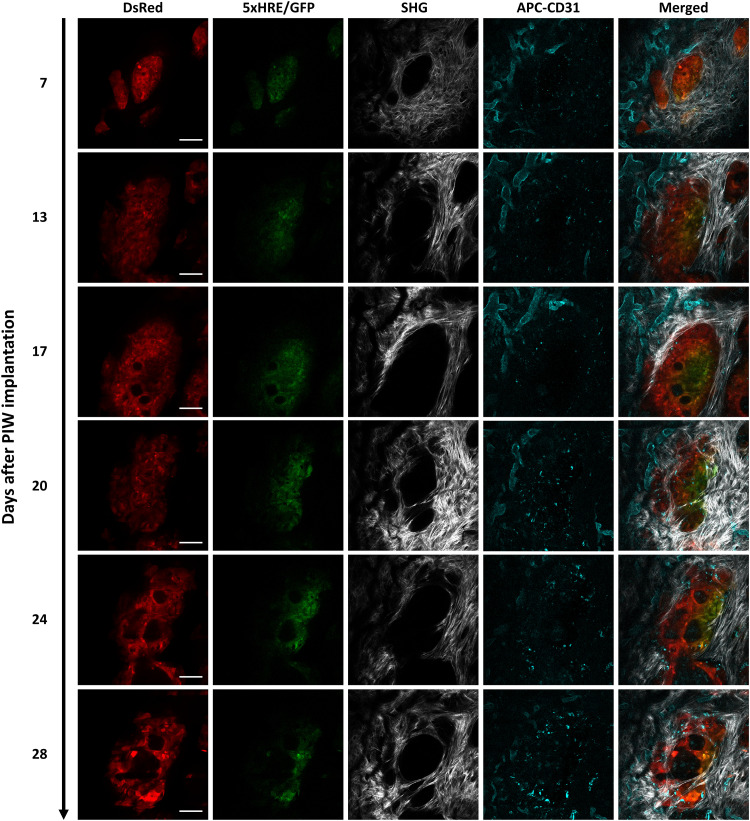
Longitudinal intravital images of an orthotopic BxPC3-DsRed-5xHRE/GFP tumor region of interest (ROI), up to 28 days after PIW implantation. BxPC3 cells (DsRed) are shown in red, HIF activity (5xHRE/GFP) in green, blood vessels (APC-CD31) in cyan, and fibrillar collagen (SHG) in white. Scale bars, 100 μm.

**Fig. 5.**
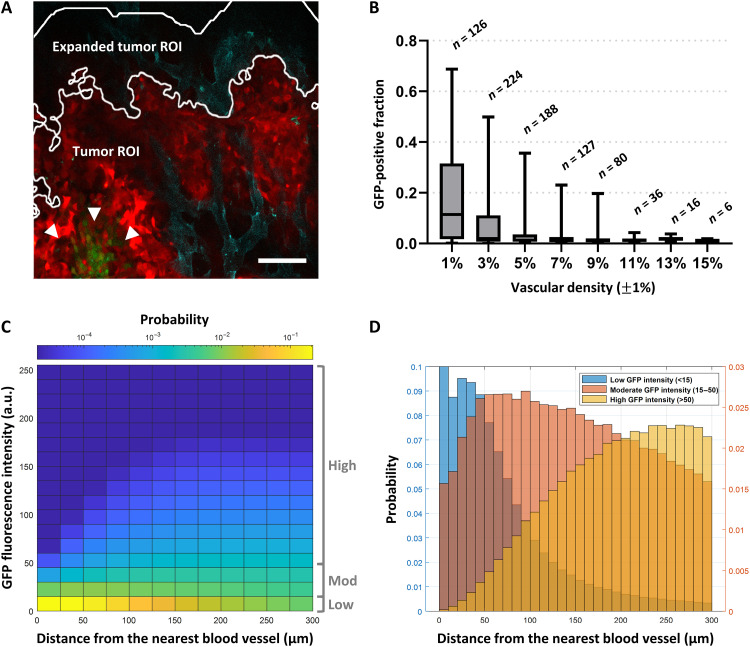
Quantitative intravital fluorescence imaging reveals an inverse relationship between tumor cell 5xHRE/GFP expression and vascular density. (**A**) An example intravital fluorescence image shows BxPC3-DsRed cells in red, GFP fluorescence (HIF activity) in green (arrowheads), and APC-CD31 (blood vessels) in cyan. The tumor ROI outlines the analysis region for BxPC3-DsRed–positive tumor pixels. The expanded tumor ROI outlines a concentric analysis region, expanded radially by 100 μm, to include blood vessels along the tumor periphery. Scale bar, 100 μm. (**B**) Aggregate data from 861 intravital fluorescence images (2429 μm by 2429 μm) of BxPC3 tumors in vivo show an inverse relationship between tumor vascular density (%) and GFP-positive fraction of tumor ROIs. *r* = −0.39, *P* < 0.0001. (**C**) A bivariate probability histogram from aggregate tumor cell fluorescence data reveals a positive relationship between BxPC3 GFP fluorescence intensity and distance to the nearest blood vessel (*r* = 0.48, *P* < 0.0001). Bins are colored from blue to yellow, indicating low to high probability within the dataset, respectively. (**D**) Grouping BxPC3 GFP fluorescence intensity (“low,” “moderate,” and “high”) demonstrates its relationship with the distance of the BxPC3 tumor cells to the nearest blood vessel. Probabilities for low GFP intensity are plotted on the left *y* axis, and probabilities for moderate/high GFP intensity are plotted on the right *y* axis.

To investigate BxPC3 5xHRE/GFP fluorescence, in vivo IVFM was performed by combining an orthotopic mouse model of pancreatic cancer with a custom-designed PIW (Fig. 2). Using this animal model, we were able to serially and simultaneously visualize BxPC3 cells (DsRed), BxPC3 HIF activity (5xHRE/GFP), blood vessels (APC-CD31), and fibrillar collagen (SHG) in real time, at cellular resolution (Figs. 3 and 4). To understand how blood vessel density affected 5xHRE/GFP expression in our tumor model, we decided to investigate this relationship across all images that were taken (*n* = 861). From these data, we found a negative correlation between tumor vascular density and GFP-positive fraction of tumor regions of interest (ROIs) [correlation coefficient (*r*) = −0.39, *P* < 0.0001; Fig. 5B]. By analyzing the distance of each tumor cell to the nearest blood vessel and aggregating these values across all tumor ROIs (*n* = 1,783,797 cells), we found a positive relationship between each tumor cell’s GFP fluorescence intensity and its distance to the nearest blood vessel (*r* = 0.48, *P* < 0.0001). This was visualized using a bivariate histogram (Fig. 5C) with the probability of each tumor cell’s GFP fluorescence intensity increasing with its distance to the nearest blood vessel. “Low” GFP fluorescence intensity (empirically determined to be below background fluorescence) was largely localized to distances less than 100 μm, while “high” GFP fluorescence intensity was localized to distances greater than 100 μm (Fig. 5D).

### BxPC3 5xHRE/GFP expression colocalizes with histological markers of tumor hypoxia

**Fig. 6.**
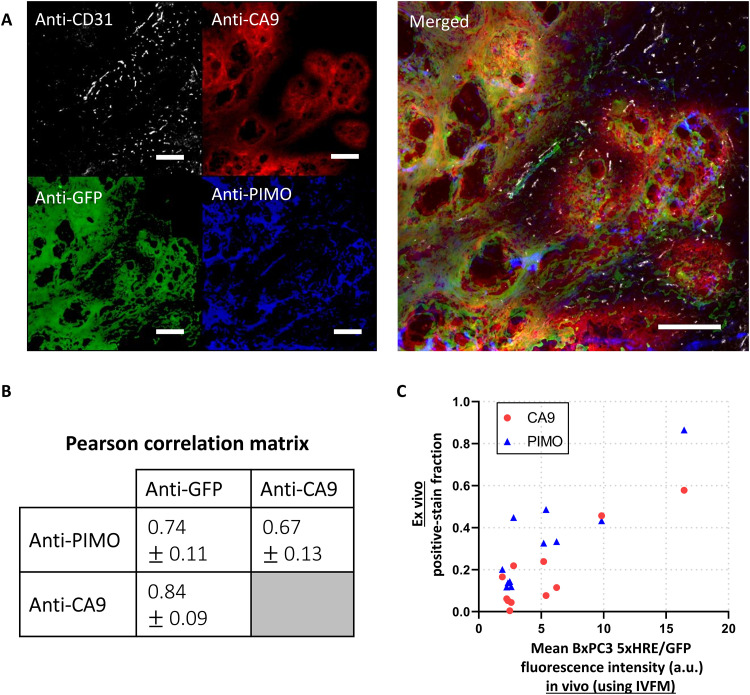
In vivo expression of 5xHRE/GFP in BxPC3 tumor cells correlates with conventional biomarkers of tumor hypoxia. (**A**) Representative immunofluorescence (IF) images from serial BxPC3 pancreatic tumor tissue sections (5 μm thick). Tumors were excised immediately after the final in vivo imaging time point. Sections were stained with anti-CD31, anti-CA9, anti-GFP, and anti-PIMO. Scale bars, 200 μm. (**B**) A correlation matrix shows the mean Pearson correlation coefficients between each IF stain, across 10 tumor samples (± SD). (**C**) A scatter plot shows the relationship between the hypoxic biomarker (CA9 and PIMO) IF positive-stain fraction in ex vivo tumor tissue sections versus the mean GFP fluorescence intensity of the same tumors in vivo, using IVFM. *r* = 0.88 (*P* = 0.0003) and 0.89 (*P* = 0.0003), respectively. *n* = 11 tumors.

The correlation between BxPC3 cell 5xHRE/GFP expression and other known biomarkers of tumor hypoxia [pimonidazole and carbonic anhydrase IX (PIMO and CA9, respectively)] was assessed using 10 different ex vivo tumor samples, each serially sectioned (5 μm thick) and immunofluorescence-stained against GFP, PIMO, and CA9 (Fig. 6A). Pixel intensity–based colocalization analysis (*33*, *34*) across each of these tumor sections found that anti-GFP stain fluorescence intensity had the strongest correlation with anti-CA9 stain fluorescence intensity, with a mean Pearson $\bar{r}$ of 0.84 (SD = 0.09). Anti-PIMO and anti-GFP stains had an $\bar{r}$ of 0.74 (SD = 0.09), while anti-PIMO and anti-CA9 stains had an $\bar{r}$ of 0.66 (SD = 0.13) (Fig. 6B).

### Intravital 5xHRE/GFP fluorescence is representative of tumor hypoxic fraction

To assess whether BxPC3 5xHRE/GFP fluorescence intensity measured using IVFM is representative of hypoxia throughout the tumor, we compared the mean tumor cell GFP fluorescence intensity in vivo (using IVFM) to the tumor hypoxic fraction from 11 different tumors, immunofluorescence-stained for both PIMO and CA9 (Fig. 6C). The mean in vivo GFP fluorescence intensity from IVFM was found to strongly correlate with the positive ex vivo stain fraction of PIMO (*r* = 0.89, *P* = 0.0003) and CA9 (*r* = 0.88, *P* = 0.0003). These relationships indicate that the mean BxPC3 5xHRE/GFP fluorescence intensity is representative of hypoxia throughout the tumor.

### Relationship between BxPC3 5xHRE/GFP fluorescence and tumor-associated fibrillar collagen

**Fig. 7.**
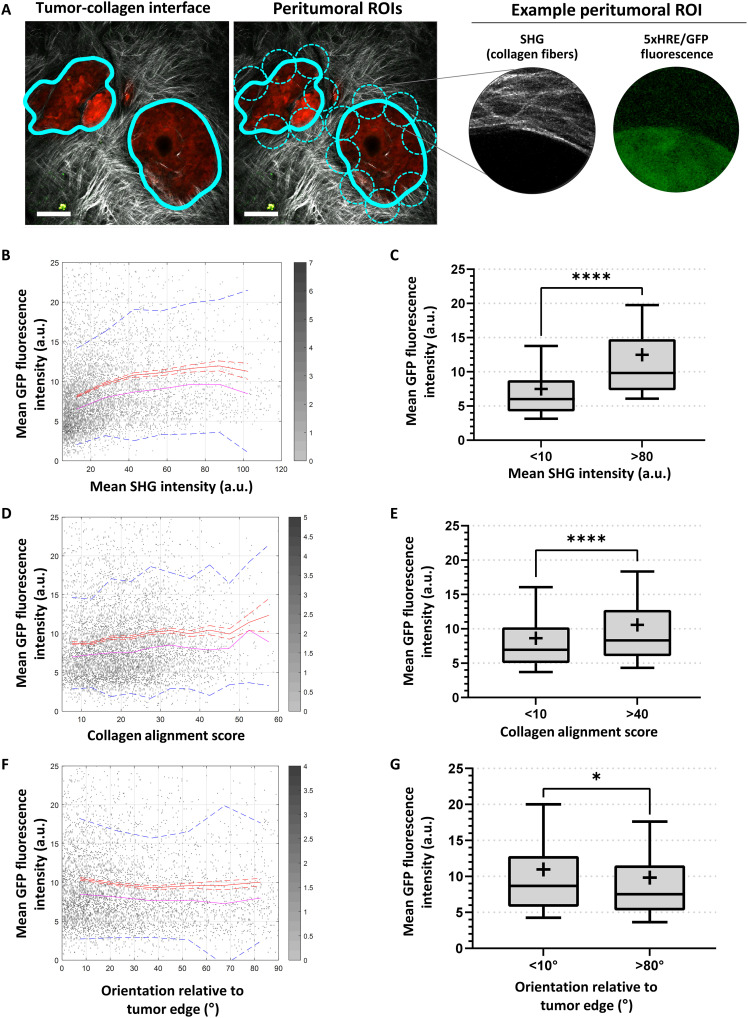
Peritumoral collagen production is associated with BxPC3 5xHRE/GFP expression. (**A**) Image analysis framework used to analyze collagen and BxPC3 5xHRE/GFP expression along the tumor-collagen interface (thick cyan line). Dashed cyan circles indicate each peritumoral ROI, with a diameter of 100 μm, spaced 100 μm apart. Scale bars, 100 μm. (**B**, **D**, and **F**) Scatter plots show the mean GFP fluorescence intensity versus mean SHG intensity (*r* = 0.21, *P* < 0.0001), alignment score (*r* = 0.09, *P* < 0.0001), and orientation relative to tumor edge (*r* = −0.05, *P* < 0.0001), respectively. *n* = 6846. The red lines indicate mean values, red dashed lines are SEM, pink lines are median values, and blue dotted lines show SD. (**C**, **E**, and **G**) Box plots show the mean GFP fluorescence intensity of BxPC3 cells for different ranges of SHG intensity, alignment score, and collagen orientation relative to tumor edge, respectively. Mean values are indicated by the “+” symbol on each boxplot. Whiskers show 10th to 90th percentile of mean GFP fluorescence intensity in peritumoral ROIs. *P < 0.05 and ****P < 0.0001.

To investigate the relationship between 5xHRE/GFP fluorescence and tumor-associated collagen in our BxPC3 tumor model, fibrillar collagen structures were visualized using in vivo SHG microscopy and analyzed along the tumor-collagen interface. A circular, moving “peritumoral ROI” with a diameter of 100 μm was used to compare local GFP fluorescence intensity with three different collagen features: mean SHG intensity, collagen fiber alignment, and collagen orientation (relative to the tumor edge) (Fig. 7A). By aggregating these measurements for all peritumoral ROIs across all images (*n* = 6846), we found a weak positive correlation between the mean GFP fluorescence intensity and mean SHG intensity (*r* = 0.21, *P* < 0.0001; Fig. 7B). Peritumoral ROIs with a high mean SHG intensity were found to have significantly higher mean GFP fluorescence intensity than peritumoral ROIs with a low mean SHG intensity (12.50 versus 7.50, *P* < 0.0001; Fig. 7C). GFP fluorescence intensity was also found to have a weak positive Pearson *r* with alignment score of collagen fibers (*r* = 0.09, *P* < 0.0001; Fig. 7D). Peritumoral ROIs with a high alignment score were found to have a higher mean GFP fluorescence intensity than peritumoral ROIs with a low alignment score (10.57 versus 8.63, *P* < 0.0001; Fig. 7E). Last, GFP fluorescence intensity was found to have a weak negative correlation with the orientation of collagen fibers relative to the tumor edge (*r* = −0.05, *P* < 0.05; Fig. 7F). Peritumoral ROIs where collagen fibers were perpendicular to the tumor edge were found to have a lower mean GFP intensity than peritumoral ROIs with collagen fibers running parallel to the tumor edge (10.96 versus 9.82, *P* < 0.0001; Fig. 7G).

## DISCUSSION

The pancreatic TME is characterized by hypoxia and desmoplasia, both of which play a major role in affecting treatment response (*9*–*13*). Accumulating evidence suggests that understanding the complex interactions between components of the pancreatic TME is necessary to identify new therapeutic targets and improve treatment strategies (*35*). However, there are several challenges in studying these interactions in vivo due to substantial heterogeneity, both within the TME and between different tumor samples (*25*–*27*). In addition, the pancreatic TME is constantly changing in composition and structure as cancer cells proliferate and invade the surrounding tissues (*25*) and in response to therapeutic interventions (e.g., chemo- and radiotherapy) (*36*). While preclinical studies are invaluable in studying how the pancreatic tumors respond to therapy, existing in vivo animal models of pancreatic tumors are limited. In this study, we undertook the development and validation of a novel intravital imaging model to study pancreatic tumors in vivo and quantitatively image tumor cells, stroma, microvasculature, and hypoxia simultaneously and in real time within the pancreatic TME.

To reliably monitor tumor cell hypoxia in vivo, we developed a dually fluorescent BxPC3-DsRed-5xHRE/GFP cell line. This human pancreatic cancer cell line constitutively expresses red fluorescent protein (DsRed) and GFP, the latter being driven by the 5xHRE/GFP reporter under hypoxia. It should be noted, however, that this model does not directly measure partial pressure of oxygen (or pO_2_) but rather the tumor cell transcriptional response to hypoxia in the microenvironment, characterized by increased tumor cell HIF activity. While it is well established in the literature that HIFs drive cellular protein expression under hypoxic conditions by binding to the HRE promoter (*37*), the time scale of different HIF transcription complexes (e.g., HIF-1 and HIF-2) in regulating cellular hypoxic response is an active area of investigation (*38*). Nevertheless, using in vitro live-cell fluorescence microscopy (Fig. 1B), we confirmed that 5xHRE/GFP is a sensitive and reliable fluorescent marker of BxPC3 cell hypoxia and was tested in cell culture for up to 4 days under 0.2, 1, and 21% O_2_. Our data show that HRE-driven GFP fluorescence intensity increases with both the duration and severity of low oxygen conditions, although this reporter may not be able to distinguish between severe, acute hypoxia and moderate, chronic hypoxia. We also show that this fluorescent reporter is reversible, with GFP fluorescence intensity returning to baseline levels within 8 to 12 hours of reoxygenation (half-life of ~2 hours; Fig. 1D). GFP was still detected using Western blot for up to 2 days after reoxygenation (Fig. 1, E and F), which is consistent with the previously reported degradation half-life of GFP (approximately 26 hours) (*39*). The difference between these two measurements is likely due to the fact that fluorescence imaging can only detect mature GFP, while Western blot assay can detect both premature and mature GFP (*40*). It is also possible that the GFP fluorescence intensity in tumor cells decreased at a faster rate than the previously reported degradation half-life of GFP due to proteins being diluted through cell division (*41*). Nevertheless, the temporal behavior of BxPC3 cell 5xHRE/GFP fluorescence suggests that fluorescence microscopy is a suitable technique for detecting cells undergoing chronic hypoxia (on the order of hours to days).

To validate the expression of 5xHRE/GFP as a marker of BxPC3 tumor cell hypoxia in vivo, we performed immunofluorescence staining on 10 individual BxPC3 tumor samples to correlate GFP expression with conventional markers of tumor hypoxia (i.e., PIMO and CA9). It is well established that hypoxic conditions in tumor cells induce CA9 expression through the HIF transcription factor, which binds to the HRE site in the CA9 promoter (*42*, *43*). Because 5xHRE/GFP and CA9 are driven by the same promoter, it is not unexpected that their cellular expression is strongly correlated with one another, with a mean Pearson $\bar{r}$ of 0.84 (SD = 0.09) across 10 tumor samples. The weaker correlations measured between 5xHRE/GFP and PIMO ($\bar{r}=0.74$, SD = 0.11) and CA9 and PIMO ($\bar{r}=0.67$, SD = 0.13) are likely due to the differences in each marker’s sensitivity to low oxygen conditions. PIMO is typically reduced at oxygen tensions less than 10 mmHg (~1% O_2_) (*44*), while CA9 is generally expressed at oxygen tensions less than 20 mmHg (~3% O_2_) (*45*). Because CA9 has a higher oxygen threshold than PIMO, it is not unexpected that we observed CA9 staining closer to tumor blood vessels (Fig. 6A). The differences in oxygen sensitivity between these markers, although subtle, can be used to measure the relative severity of tumor hypoxia. Healthy tissue oxygen levels range between 3 and 7% O_2_ (*46*). Once oxygen falls below this range [termed “physiological hypoxia”; (*47*)], several physiological processes begin to respond to maintain oxygen homeostasis, e.g., vasodilation, increasing blood flow, and up-regulation of hypoxia response genes (primarily driven by HIF) (*48*). “Pathological hypoxia,” defined as oxygen levels below 8 mmHg (1% O_2_) (*47*), suggests a disruption to normal oxygen homeostasis resulting in the persistence of poor oxygenation. While PIMO is a more suitable marker for pathological hypoxia in tissues and, as an exogenous molecule, provides more dynamic measurements of pO_2_ (*49*), it does not provide any information regarding each tumor cell’s biological response to hypoxia. In contrast, endogenous biomarkers CA9 and 5xHRE/GFP provide direct measurements of the cellular transcriptional response to hypoxia, and we have shown in vitro that tumor cell GFP expression responds dynamically to changing oxygen conditions (Fig. 1C). Future studies should explore the dynamics of tumor cell 5xHRE/GFP expression in experimental orthotopic tumors using compounds such as carbogen and hydralazine to modulate tumor hypoxia in vivo in mice (*49*). Nevertheless, the data presented here demonstrate that 5xHRE/GFP expression can measure the relative magnitude of hypoxic stress experienced by BxPC3 tumor cells in vivo.

Within the pancreatic TME, the lack of functional vasculature plays an important role in the establishment and sustained presence of tumor hypoxia (*50*). Using IVFM, we observed an inverse relationship between the vascular density and the hypoxic (GFP-positive) fraction of BxPC3 cells (*r* = −0.39, *P* < 0.0001; Fig. 5B). This is not unexpected because hypovascular tumor regions typically have less access to oxygen, making cells more susceptible to hypoxic stress (*50*, *51*). Furthermore, we demonstrate the spatial relationship between blood vessels and BxPC3 HIF activity by analyzing the distance transform of tumor blood vessels to tumor cells (Fig. 5, C and D). We consistently observed a spatial gradient of BxPC3 5xHRE/GFP fluorescence intensity that increases with the distance of the BxPC3 cells from blood vessels (*r* = 0.48, *P* < 0.0001). This is consistent with previously observed “hypoxia gradients” in tumor tissues (*52*, *53*). However, given that our study analyzed intravital images from specific ROIs, we may have excluded blood vessels outside the field of view and optical depth of field. Thus, it is possible that our BxPC3 cell distance–to–nearest blood vessel measurements overestimated the actual Euclidean distance between pancreatic tumor cells and their nearest blood vessel. In addition, because the microvasculature of tumors is known to be irregular and dysfunctional (*54*, *55*), the presence of blood vessels in the fluorescence images may not necessarily mean that sufficient levels of oxygen are being delivered to those tissues. Nevertheless, this intravital imaging model can be used to measure oxygen diffusion gradients in pancreatic tumors in vivo and potentially study its effects on tumor progression. Oxygen gradients have been shown to influence macrophage recruitment, which plays a critical role in cancer progression (*56*). In addition, the slope of the spatial oxygen diffusion gradient (as a function of distance from blood vessels; Fig. 5, C and D) may be used to study changes in metabolic oxygen consumption rates (*53*) of cancer cells over time or in response to treatment (such as radiotherapy). For example, shorter oxygen gradients from blood vessels could indicate that tumor cells may have higher oxygen consumption (*57*) and are actively proliferating. Longer oxygen gradients may indicate that the cancer cells have lower oxygen consumption rates, becoming tolerant to hypoxia in the TME, affecting overall malignancy and treatment efficacy (*58*, *59*). Thus, our quantitative intravital imaging model may serve as a platform for future investigation of tissue oxygen gradients within the pancreatic TME and their spatiotemporal relationship to tumor blood vessels, immune cells (e.g., macrophages), and cellular oxygen consumption rates in vivo.

Clinical findings indicate that one of the most prominent features of pancreatic tumors is their highly desmoplastic TME (*25*, *35*, *60*, *61*). This desmoplasia creates a physical barrier around the tumor (*35*), which can alter blood flow resulting in hypoxia (*62*), decreased drug delivery (*16*), and immune infiltration (*63*), ultimately increasing resistance to chemotherapy (*16*), radiotherapy (*64*, *65*), anti-angiogenic therapy (*66*), and immunotherapy (*63*). Previous studies have shown that pancreatic tumor–associated collagen can induce tumor hypoxia and vice versa (*21*). Using our model, we can image tumor-associated collagen in vivo using intravital SHG microscopy. By analyzing fibrillar collagen surrounding the tumor from SHG images, we found a weak positive relationship between the mean 5xHRE/GFP fluorescence intensity of tumor cells and mean SHG intensity (*r* = 0.21, *P* < 0.0001; Fig. 7). These findings are consistent with previous studies showing that tumor-associated collagen coincides with hypoxic regions within tumors (*50*, *67*–*69*). Several studies have demonstrated that hypoxia modifies tumor ECM components, increasing collagen deposition in the TME as well as ECM density and stiffness (*70*–*72*). Both cancer cells and cancer-associated fibroblasts are known to contribute to ECM remodeling in response to hypoxia (*72*, *73*). Conversely, a desmoplastic stroma contributes to tumor hypoxia by displacing capillaries with high amounts of ECM, further reducing the supply of oxygen and perpetuating a cycle leading to even more hypoxia and metabolic stress (*20*, *74*–*78*). Nevertheless, there is still controversy in the literature about whether the fibrotic reaction in the pancreatic TME ultimately promotes or inhibits disease progression (*24*, *79*). Our model may facilitate the study of the dynamic interactions between tumor hypoxia and desmoplasia and how this relationship affects tumor growth, metastasis, or treatment response.

In addition to an increase in collagen production in the TME, the spatial alignment of collagen fibers around the tumor was also found to be related to BxPC3 5xHRE/GFP fluorescence intensity (*r* = 0.09, *P* < 0.0001; Fig. 7, D and E). This relationship is consistent with previous studies showing that, through multiple mechanisms, hypoxic fibroblasts generate and organize highly aligned ECM (*80*–*82*). However, given that the relationship between hypoxia and collagen alignment was relatively weak in our dataset, it is still unclear what other factors contribute to collagen alignment in the pancreatic TME and how this ultimately affects disease progression. Drifka *et al.* (*83*) have shown that spatially aligned stromal collagen is a negative prognostic factor in pancreatic cancer, associated with poor survival. In contrast, Bolm *et al.* (*84*) have shown that pancreatic tumors with randomly oriented stromal fibers were associated with larger tumor size, nodal-positive disease, margin-positive resection rates, and poorer overall survival. This inconsistency in the literature suggests that a more detailed, mechanistic understanding is needed to uncover how the spatial alignment of collagen fibers in the pancreatic TME affects tumor progression. Using our intravital model of pancreatic cancer, we can continue to explore what factors contribute to collagen fiber alignment and how this may have downstream effects on pancreatic tumor growth, remodeling of the TME, and metastasis.

We also found that collagen fibers oriented parallel (<10°) to the tumor boundary were weakly associated with increased local BxPC3 5xHRE/GFP fluorescence intensity, especially when compared to tumor regions with collagen fibers oriented perpendicular (>80°) to the tumor boundary (Fig. 7G). Collagen fiber orientation has been previously shown to steer both angiogenesis and endothelial cell migration (*85*, *86*). Thus, it is possible that radially oriented collagen fibers (perpendicular to the tumor boundary) may allow blood vessels to more easily migrate into the tumor and increase tumor oxygenation. Emerging evidence suggests that radially oriented collagen fibers can also provide avenues for tumor cells to invade the surrounding tissues to escape their hostile microenvironment (*87*). Conversely, tangentially oriented collagen fibers (parallel to the tumor boundary) may be confining tumor cells as they continue to proliferate, causing an increase in solid stress and interstitial fluid pressure (*88*, *89*). As desmoplasia progresses in pancreatic tumors, an increase in solid stress can contribute to reduced vascular patency along the tumor boundary, leading to increased tumor hypoxia (*77*, *90*). This leads to a vicious cycle—whereby tumor growth leads to solid stress, solid stress leads to hypoxia, hypoxia leads to collagen remodeling (*91*), and collagen remodeling influences angiogenesis and tumor cell invasion—further driving tumor growth and disease progression. Remodeling of the tumor ECM along the tumor boundary is an ongoing and dynamic process (*92*). Future studies can potentially benefit from using our pancreatic intravital imaging model to study the relationship between tumor hypoxia and ECM remodeling, which collectively influence tumor cell invasion and metastasis (*80*). The successful development of novel treatments targeting the TME stromal compartment depends on a more complete understanding of these tumor-stromal interactions.

Together, this study presents a key advancement over conventional methodologies of studying the pancreatic TME and previously adopted methods of intravital imaging in pancreatic tumor models. By combining multispectral IVFM and SHG microscopy with a custom-desigend, surgically implanted imaging window, we were able to consistently and simultaneously image pancreatic cancer cells, tumor microvasculature, and fibrillar collagen at cellular resolution in vivo, for up to 4 weeks. We also developed and validated a novel, dually fluorescent pancreatic DsRed-5xHRE/GFP reporter cell line for real-time monitoring of tumor hypoxia in the orthotopic setting. In this study, we used the 5xHRE/GFP reporter to quantify tumor cell hypoxia and hypoxic gradients in the orthotopic in vivo tumor setting and postulated a potential means of measuring oxygen consumption rates of tumor cells relative to tumor microvasculature. Last, we used this model to quantify the spatial relationship between tumor cell hypoxia and tumor-associated collagen structures and hypothesized potential mechanisms by which collagen fiber density, alignment, and orientation could affect tumor hypoxia, growth, invasion, metastasis, and treatment response.

While cell line–derived tumor models, such as the one used in this study, are ideal for intravital imaging experiments due to their consistent and predictable tumor growth kinetics, they are limited in their ability to study the TME due to their severely immunodeficient host system. Thus, future work should continue to build on this fluorescent reporter model, adapting it to new cell lines to show broad utility of this reporter system, as well as immunocompetent GEMMs that can better recapitulate the pancreatic TME in human disease. This could substantially enhance the clinical translational aspect of such research. By combining these emerging technologies with the intravital imaging platform described in this work, we can improve our understanding of the biological processes that promote pancreatic cancer progression and help us identify mechanisms of treatment resistance within the pancreatic TME.

## METHODS

### Cell line and live-cell fluorescence microscopy

Fluorescently labeled BxPC3-DsRed human pancreatic adenocarcinoma cells (AntiCancer, San Diego, CA, USA) (*93*) were grown in RPMI 1640 medium supplemented with 2 mM l-glutamine, 10% fetal bovine serum, and 1% penicillin-streptomycin at 37.0°C and 5% CO_2_. To report on tumor cellular HIF activity, BxPC3 cells were stably transfected with a 5xHRE-GFP construct [“5xHRE/GFP” (Fig. 1A), a gift from M. Brown and T. Foster; Addgene, plasmid no. 46926], as previously described (*94*). The GFP expression of this modified cell line, hereinafter referred to as BxPC3-DsRed-5xHRE/GFP, was characterized in vitro at various oxygen concentrations (0.2, 1.0, and 21% O_2_) using an H45 HEPA HypOxystation (HypOxygen Inc., Frederick, MD, USA). Cells were seeded in 12-well plates at 15% cell confluence and placed in a normoxic (21% O_2_) incubator for 24 hours. Cell plates were then transferred to incubators at each respective oxygen concentration, for up to 4 days (reaching approximately 80% cell confluence). Live-cell fluorescence imaging was performed at intermittent (daily/hourly) time points using a Zeiss AxioObserver Z.1 inverted microscope (Carl Zeiss Ltd., Toronto, Canada) with a large chamber microscope incubator (Pecon GmbH, Erbach, Germany).

### Western blot

To confirm and quantify the relative expression of GFP, BxPC3-DsRed-5xHRE/GFP cell lysates were collected at several time points under either hypoxia (0.2% O_2_) or normoxia (21% O_2_). Cells were washed with ice-cold phosphate-buffered saline and harvested in radioimmunoprecipitation assay (RIPA) buffer and protease inhibitor cocktail (BioShop, diluted 1:100 in RIPA buffer). Cell lysates were incubated for 1 hour on ice (vortexed every 30 min). The soluble fractions of cell lysates were isolated by centrifugation at 11,000*g* for 20 min (4°C), and a bicinchoninic assay (Pierce BCA Protein Assay Kit, Thermo Fisher Scientific) was used to normalize protein concentrations. Cell lysates were diluted 1:1 in 2x Laemmli buffer, and gel electrophoresis and membrane blotting were performed using equipment purchased from Bio-Rad Laboratories (CA, USA). Polyvinylidene fluoride membranes were incubated for 24 hours at 4°C in an antibody solution (see Table 1 for antibody and dilutions used). All membranes were scanned using the LI-COR Odyssey Imaging System and analyzed in Empiria Studio software (LI-COR Biosciences Inc.). Band intensity was quantified using Fiji ImageJ software (*95*).

**Table 1.** Antibodies and dilutions used in the study. N/A, not applicable.

	Primary antibody	Company, catalog no.	Dilution	Secondary antibody/reagent	Company, catalog no.	Dilution
Western blot	Anti-GFP	Novus Biologicals, NB600-308	1:2000	IRDye 680RD Detection Reagent	LI-COR Biosciences, 926-69100	1 μl/ml
	Anti–β-tubulin	MilliporeSigma, 05-661	1:10,000			
IVFM	APC-conjugated anti-CD31	BD Biosciences, 551262	0.03 mg/ml	N/A		
Tissue immunofluorescence	Anti-CD31	BD Biosciences, 550274	1:100	Anti-rat Alexa Fluor 488	Thermo Fisher Scientific, A-11066	1:200
	Anti-PIMO	Hypoxyprobe, Pab2627	1:100	Anti-rabbit Alexa Fluor 555	Thermo Fisher Scientific, A-21428	1:200
	Anti-CA9	Novus Biologicals, NB100-479SS	1:50	Anti-rabbit Alexa Fluor 555	Thermo Fisher Scientific, A-21428	1:200
	Anti-GFP	Novus Biologicals, NB600-308SS	1:500	Anti-rabbit Alexa Fluor 555	Thermo Fisher Scientific, A-21428	1:200

### Mouse model and PIW surgery

All animal experiments were conducted in accordance with regulatory standards approved by the University Health Network Animal Care Committee (Animal Use Protocol no. 2613). Orthotopic BxPC3-DsRed-5xHRE/GFP pancreatic tumors were established in 8- to 10-week-old female NOD-Rag1^null^ IL2rg^null^ (NRG) mice (Jackson Laboratory, no. 007799) as previously described (*96*). Once tumors reached ~5 mm in diameter (5 weeks after inoculation), a custom-designed PIW, with an inner diameter of 12 mm, a thickness of 1.8 mm, and eight equally spaced 1-mm holes (Fig. 2, A and B), was three-dimensionally (3D) printed with acrylonitrile butadiene styrene plastic and surgically implanted over the tumor site (Fig. 2C). The surgical procedure for PIW implantation was performed as previously described (*97*), with modifications. Briefly, anesthetized animals received buprenorphine (0.1 mg/kg) and were maintained with 2% isoflurane in oxygen in right-lateral position on a heated pad. Fur was removed on the left lateral side, and the surgical site was prepared with iso-betadine and ethanol solutions. A 15-mm paramedian incision was made in the left flank of the mouse, above the pancreas. The PIW was positioned under the skin and interrupted, and 4-0 nonabsorbable sutures (Prolene, Ethicon) were placed in each hole around the PIW, securing it to the skin. A lobe of the pancreas (containing the pancreatic tumor) was extracorporated through the center of the PIW using sterile cotton swabs. One to two interrupted, 4-0 absorbable sutures (Polysorb, Covidien) were placed in the abdominal muscle to prevent the tumor from retracting back into the abdominal cavity. A #2 glass microscopic coverslip (Electron Microscopy Sciences, no. 72226) was inserted in the PIW and kept in place with a plastic snap ring. Mice were given 7 days to recover before imaging. In total, 33 animals were used in this study.

### Intravital fluorescence and SHG microscopy

After 1 week of recovery from PIW implantation, serial intravital fluorescence and SHG imaging of pancreatic tumors was performed using a LSM710 laser scanning confocal microscope (Carl Zeiss Canada Ltd.; Fig. 2D). To facilitate live-animal imaging on the microscope and obtain consistent images, a custom-designed, 3D printed microscope stage insert was used with a built-in isoflurane port to maintain anesthesia, an electrical heating pad to maintain physiological body temperature, and a PIW holder to reduce motion artifacts while imaging (Fig. 2E). All animals were anesthetized using isoflurane in oxygen (5% induction and 2% maintenance) and kept at 37°C throughout each imaging session. Tumor blood vessels were fluorescently labeled via intravenous injection of 6 μg of APC-conjugated rat anti-mouse CD31 antibody (see Table 1). Lasers at 488 and 633 nm were used to excite GFP/DsRed and APC, respectively. An 840-nm laser was also used to create an SHG signal for visualization of fibrillar collagen within the pancreatic tumors in vivo.

In total, 33 mice were subject to intravital imaging. Mice were imaged every 3 to 5 days for up to 28 days after PIW implantation. This was previously determined to be a reliable period for imaging before the PIW was dislodged from the abdominal wall or skin (*98*). For each mouse-imaging session, four unique fields of view (FOVs) within the imaging window were taken using a 5×/0.25 numerical aperture (NA) lens (FOV = 2429 μm by 2429 μm), and 10 unique FOVs were taken with a W Plan-Apochromat 20×/1.0 NA water immersion lens (FOV = 607 μm by 607 μm). All analyses involving tumor vasculature and collagen structures were performed using images collected under 5× and 20× FOVs, respectively.

### Intravital image analysis

All intravital images were analyzed using a custom MATLAB script (vR2021A, The MathWorks Inc.). Before quantification, linear unmixing of all spectral image data was performed using an algorithm previously described by Zimmerman (*99*). Briefly, a coefficient matrix (*R*) was first created using control samples of each fluorophore to measure their relative contribution to each channel. Then, the measured signal intensity in each channel (*S*) was used to determine the amount of each fluorophore (*A*) by solving matrix Eq. 1

S=A×R (1)

To quantify 5xHRE/GFP fluorescence intensity in BxPC3 cells, a binary image was first created using DsRed fluorescence intensity to identify the tumor cell ROI (“tumor ROI,” Fig. 5A). Thresholds for both DsRed and GFP fluorescence intensities were empirically determined to be above background fluorescence. The GFP-positive fraction was calculated by taking the number of GFP-positive pixels within the tumor ROI divided by the total number of pixels in the tumor ROI. Mean tumor cell GFP fluorescence intensity was calculated as the sum of each pixel’s GFP fluorescence intensity within the tumor ROI divided by the number of pixels in the tumor ROI.

To quantify tumor vasculature, the tumor ROI binary image was morphologically dilated by 100 μm beyond the tumor boundary (creating an “expanded tumor ROI,” Fig. 5A) to account for blood vessels along the tumor periphery [and within the nominal diffusion limit of O_2_ in tissues; (*100*)]. Blood vessels were segmented from APC-CD31 fluorescence intensity by performing simple linear iterative clustering (*101*) followed by adaptive thresholding (*102*). Tumor vascular density was calculated as the number of APC-positive pixels within the expanded tumor ROI divided by the total number of pixels in the expanded tumor ROI.

To analyze BxPC3 cell 5xHRE/GFP fluorescence intensity in the images, equally spaced pixels within the tumor ROI (spaced 20 μm apart, the average diameter of BxPC3 cells) were sampled to approximate individual tumor cell GFP fluorescence intensity. The distance of each tumor cell to the nearest blood vessel was determined using a distance transform (*103*) of all APC-CD31 fluorescence-positive pixels, where each cell in the tumor ROI is assigned a distance value to the nearest blood vessel wall.

To investigate the correlation between tumor-associated collagen fibers and BxPC3 5xHRE/GFP fluorescence (using the in vivo SHG and fluorescence images, respectively), a 100-μm-diameter circular, moving peritumoral ROI was analyzed every 100 μm center to center along the peritumoral border of the tumor-collagen interface (Fig. 7A). We chose 100 μm as the diameter for each peritumoral ROI as this spans the approximate limit of O_2_ diffusion in tissues (*100*). Within each peritumoral ROI, mean GFP fluorescence intensity was calculated in all pixels that overlapped with the tumor ROI (see above). Mean SHG intensity was calculated as the sum of each pixel’s SHG intensity divided by the total number of pixels. To determine the spatial alignment and orientation of collagen fibers within each peritumoral ROI, a 2D fast Fourier transform was used to generate a power spectrum, as previously described (*104*–*106*). A circular selection in the center of the power spectrum was summed radially, and the resultant data were projected onto a polar plot and fit with an ellipse. The tilt angle of the ellipse indicated the dominant “orientation” of collagen fibers within each peritumoral ROI. An identical procedure was performed on the tumor-collagen interface line to determine the orientation of the tumor edge. The difference in orientation of collagen fibers and the tumor edge was used to calculate the orientation of collagen fibers relative to the tumor edge. The ratio of minor (*b*) and major (*a*) axes of the ellipse was used to calculate the “alignment score” using Eq. 2. An alignment score of 0 indicates collagen fibers that are completely unaligned with one another, whereas a score of 100 indicates that collagen fibers are perfectly aligned

$$\text{Alignment score}=\left(1-\frac{b}{a}\right)\times 100$$ (2)

### Immunofluorescence staining and analysis of excised tumors

Twenty-eight days after PIW implantation, mice were euthanized, and whole tumors were excised from the pancreas. Tumor tissue was embedded in optimal cutting temperature compound (Tissue-Tek) and flash-frozen in liquid nitrogen. Eleven tumors were then randomly selected for immunofluorescence staining. From the center of each tumor, four serial 5-μm-thick sections were cut using a cryostat. Tissue sections were stained with antibodies for DsRed (BxPC3 tumor cells), GFP (driven by HIF activity), CD31 (endothelial cells), and hypoxia markers: PIMO and CA9. Each section was subsequently stained with a secondary antibody (see Table 1 for antibodies and dilutions used). Slides were scanned at ×20 magnification, digitalized using a whole-slide scanner (Zeiss AzioScan.Z1), and uploaded into the HALO Image Analysis software (v3.3.2541.301, Indica Labs). Consecutive images from serially sectioned tumors were registered using HALO’s Serial Stain Registration module and exported as .tiff files. All subsequent analysis was performed in MATLAB (vR2021a, The MathWorks Inc.).

To investigate the relationship between BxPC3 5xHRE/GFP expression and established biomarkers of hypoxia (PIMO and CA9), pixel intensity–based colocalization analysis (*33*, *34*) was performed on images from immunofluorescence-stained tumor tissue sections. One tissue sample was excluded from this analysis as there was no tumor hypoxia and, thus, no positive staining. Before colocalization analysis, the resolution of each image was reduced by a factor of 100 to account for errors in spatial image registration between serial sections (using MATLAB’s “resize” function with bicubic interpolation). With each resized pixel representing an area of 32.5 μm by 32.5 μm, a Pearson *r* was calculated to determine the relationship between GFP, PIMO, and CA9 staining intensities. To determine the positive stain fraction of PIMO and CA9 for each tumor, the top 10th percentile of fluorescence intensity across all stained tissues was thresholded in MATLAB to create a binary image of positively stained tissue. The total area of CA9- and PIMO-positive tissue within the area of viable tumor tissue (DsRed fluorescence positive) was divided by the total area of viable tumor tissue.

### Statistical analysis

All statistical analyses were performed using GraphPad Prism v9.3.1. For in vitro live-cell fluorescence data, a repeated-measures analysis of variance (ANOVA) followed by Dunnett’s multiple comparisons test was performed to compare multiple oxygen conditions to normoxia (21% O_2_) at each time point. For densitometric analysis of Western blot data, a one-way ANOVA followed by Dunnett’s multiple comparisons test was used to compare each condition to “0 days” under hypoxia. All correlations were determined using the Pearson *r*. Comparisons between two groups were performed using an unpaired *t* test with Welch’s correction. All data are represented as means ± SEM unless otherwise specified, with significance determined at *P* < 0.05.

## Notes

View/request a protocol for this paper from *Bio-protocol*.
